# Shield ulcer in keratoconus in the absence of atopic or vernal kerato-conjunctivitis

**DOI:** 10.3205/oc000253

**Published:** 2025-06-12

**Authors:** Magdalena Niestrata, Shuchi Kohli, Mohammad Saleki, Zahra Ashena

**Affiliations:** 1 Department of Ophthalmology, Queen’s Hospital, Romford, United Kingdom

## Abstract

Corneal shield ulcer is a severe complication of atopic keratoconjunctivitis (AKC) and vernal keratoconjunctivitis (VKC). This condition is caused by the mechanical irritation of the corneal epithelium due to giant papillae and toxic epitheliopathy resulting from inflammatory mediators. To date, there have been no reported cases of corneal shield ulcers in the literature without AKC or VKC. However, the authors have reported a unique case of shield ulcer in a patient with keratoconus but no history of AKC or VKC. The cause is hypothesised to be due to mechanical friction between the corneal steep apex and palpebral conjunctiva. Additionally, a new technique, the use of a dry amniotic membrane, is described to manage the persistent epithelial defect in shield ulcers.

## Introduction

Corneal shield ulcer is a complication of atopic and vernal keratoconjunctivitis, occurring in approximately 3–11% of patients with vernal keratoconjunctivitis (VKC), which causes significant morbidity and challenges to treatment [1], [2]. They appear as shallow greyish indolent ulcers usually located in the superior part of the cornea. The pathophysiology of corneal shield ulcers is thought to have a mechanical and inflammatory or chemical component. The mechanical friction of the sub-tarsal giant papillae causes micro-abrasions on the cornea [3]. This is coupled with active inflammation caused by chemical mediators released by eosinophils, which have cytotoxic properties and prevent epithelial healing, leading to the development of recurrent epithelial micro-erosion, adherence of mucus to the plaque and subsequent deposition of calcium in the plaque, which further prevents re-epithelialization [4]. 

Microscopy of the plaque has demonstrated collagen fibrils extending from the Bowman’s layer into the deep layers of the plaque and toxic eosinophilic granule protein in the inflammatory debris of patients with shield ulcer [5].

Treatment of shield ulcers ranges from topical and systemic medical therapy to surgical debridement of the plaque to promote re-epithelialization, depending on the severity of the ulcers [6].

In the literature, shield ulcers have been described solely in association with VKC and atopic keratoconjunctivitis (AKC), and no reports exist of its presence without these associations [6]. The authors have reported a case of a shield ulcer in a patient with keratoconus, without any allergic or atopic eye disease. They have hypothesised that the possible cause, in this case, may be due to the mechanical friction between the corneal apex and palpebral conjunctiva in a steep ectatic cornea, which triggers the formation of epithelial micro-erosions and calcium deposition, ultimately resulting in a calcific shield ulcer.

Furthermore, the authors present successful management of this condition with a novel treatment modality – a dry patch of amniotic membrane with a very good visual outcome.

## Case description

A 38-year-old Caribbean male, who had bilateral keratoconus and underwent left eye epithelium-off corneal crosslinking, using the Dresden protocol 10 years ago, presented with a one-month history of discomfort in the left eye, photosensitivity, and reduced vision. The patient’s left eye was his only functional eye due to severe vision impairment in the other eye caused by advanced keratoconus with a maximum keratometry of 79 D. He could not tolerate rigid gas-permeable contact lenses and had discontinued follow-ups for ten years. The patient had no medical history of asthma, eczema, or hay fever and denied experiencing symptoms of itchy eyes or habitual eye rubbing.

**Figure 1:**
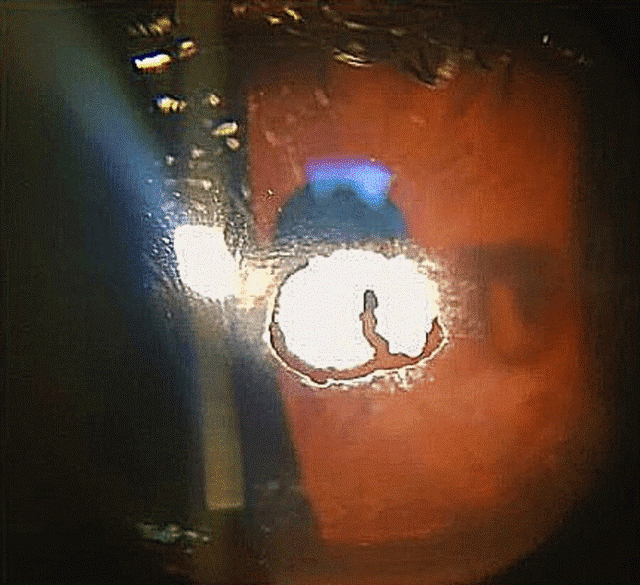
Calcified shield ulcer measuring 3 mm by 2.8 mm affecting the paracentral cornea

**Figure 2:**
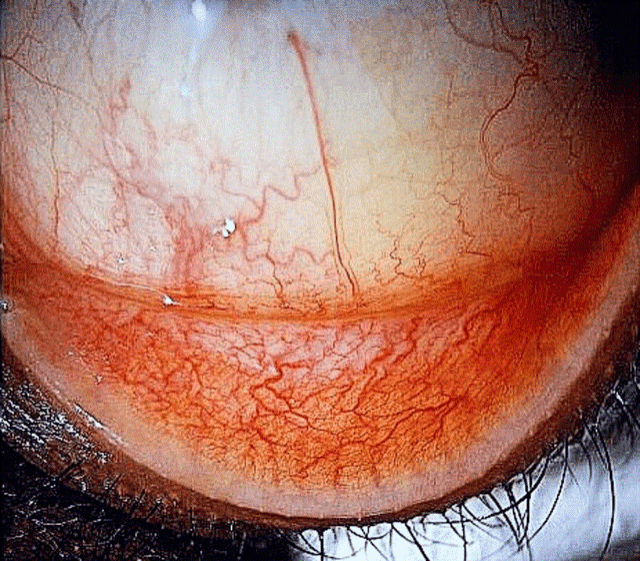
Lower tarsal conjunctiva, with no evidence of papillary reaction

**Figure 3:**
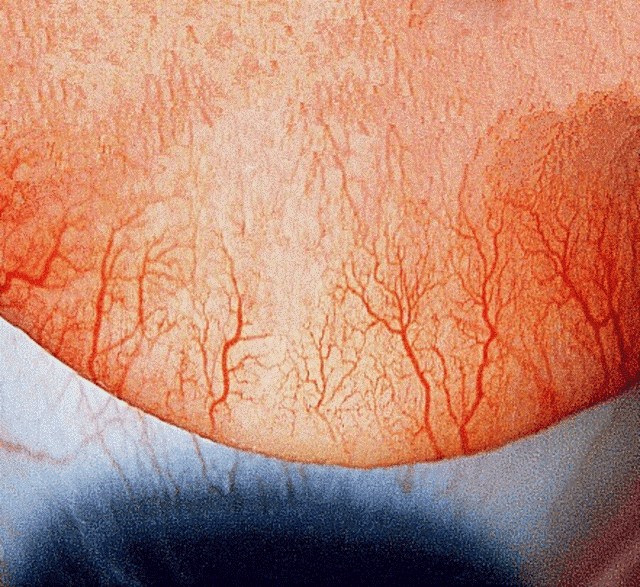
Upper tarsal conjunctiva with no giant papillae

On examination, the best corrected visual acuity (BCVA) with glasses was counting fingers in the right and 1.06 LogMAR in the left eye. The slit lamp examination showed a distinctive calcified shield ulcer, measuring 3 mm x 2.8 mm, on the paracentral cornea, with significant stromal thinning, no associated corneal neovascularization or any signs of allergic eye disease, i.e. limbal Horner-Trantas dots or palpebral micro/macro-papillae (Figure 1 (Fig. 1), Figure 2 (Fig. 2), Figure 3 (Fig. 3)).

The corneal sensation was intact. The fellow eye showed advanced keratoconus with mild apical scarring and Vogt striae and no features of allergic eye disease.

The calcific deposition was partially debrided at the slit lamp and treatment with topical preservative-free Dexamethasone 0.1% and preservative-free Chloramphenicol 0.5% was commenced. A further attempt to debride the residual calcific plaque was abandoned due to the extreme stromal thinning and the risk of corneal perforation. Despite there being no reported underlying health issues, blood tests were conducted and all results returned normal, ruling out hypercalcemia.

**Figure 4:**
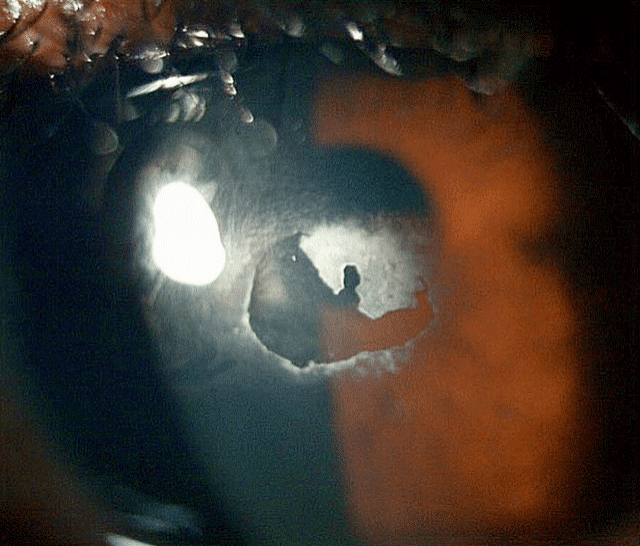
Partially removed calcific deposit and corresponding corneal epithelial defect

**Figure 5:**
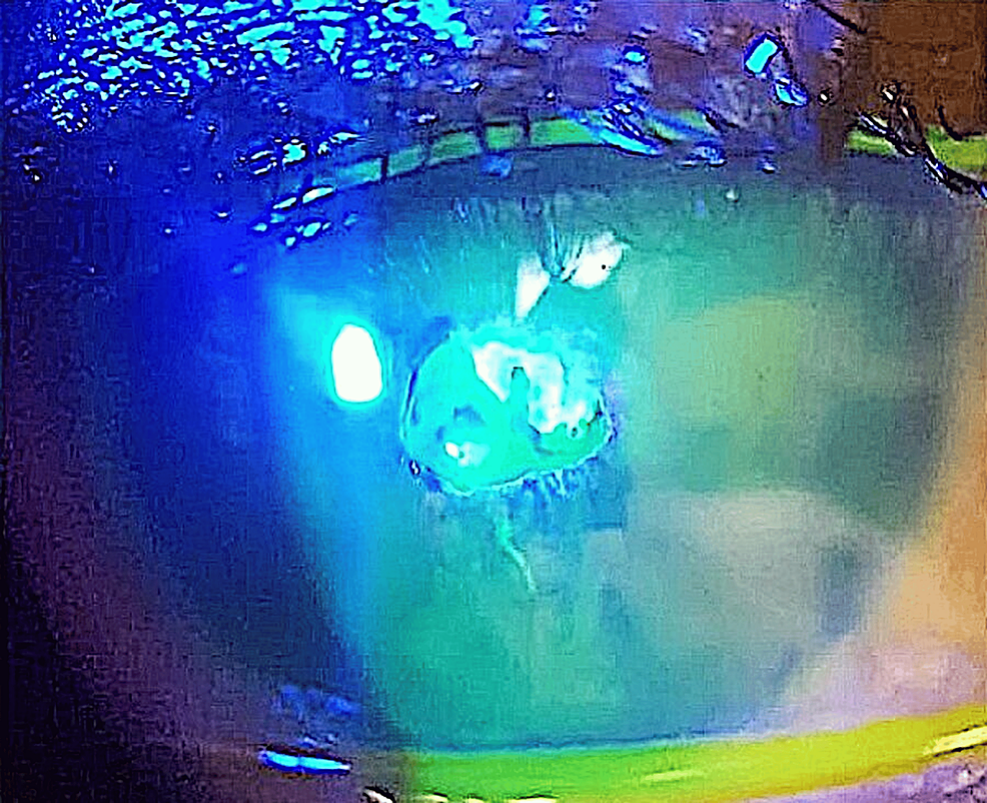
Persistent corneal epithelial defect, two weeks after partial removal of the calcific membrane and starting the medications

At two weeks follow-up, the patient remained symptomatic with continuing pain, photosensitivity and reduced vision. The cornea showed a persistent epithelial defect, measuring 2.7 mm x 2.5 mm, with a surrounding residue of plaque. Figure 4 (Fig. 4) shows partially resected calcific plaque, with persistent epithelial defect surrounded by raised margin, which prevented migration of epithelial cells from the limbus to the defect. Fluorescein staining confirmed a persistent epithelial defect in the paracentral cornea (Figure 5 (Fig. 5)).

The treatment was continued by adding preservative-free lubricating drops every two hours, and surgical debridement of the calcific plaque was planned. He underwent surgical excision of the calcific plaque and 360 degree removal of the raised margin surrounding the bare stroma, using a crescent blade. Also, to facilitate epithelialization, a dry amniotic membrane disc of 12 mm diameter was placed on the cornea (epithelial side f) and secured with a bandage contact lens (video in Attachment 1 ). The amniotic membrane used was produced by Omnigen^®^, who generate their membrane from consented mothers having planned caesarean sections.

**Figure 6:**
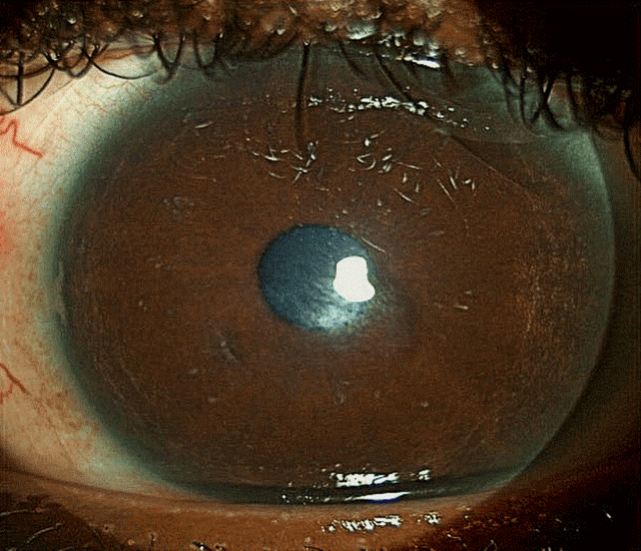
Left cornea 2 weeks post-operatively. The amnion has almost absorbed, and the bandage contact lens is in place.

One week post-operatively, the symptoms were improved, and the eye was comfortable. Examination showed a quiet eye with partially disintegrated amniotic membrane with a paracentral faint scar (Figure 6 (Fig. 6)).

**Figure 7:**
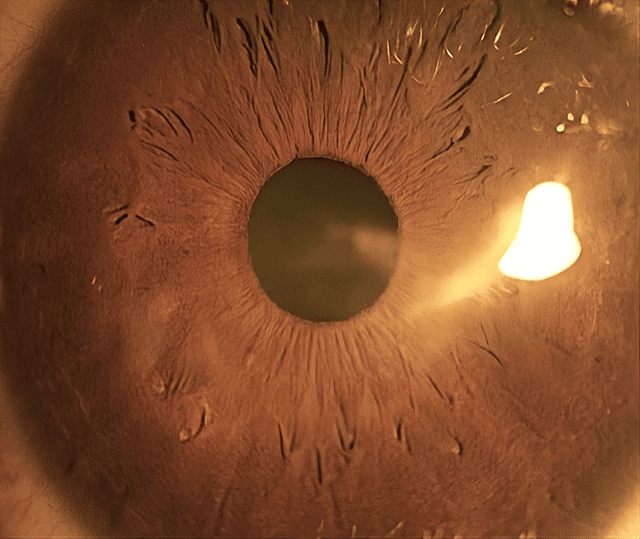
Left cornea 3 weeks post-operatively. The amniotic membrane has fully absorbed, and the bandage contact lens is removed. A faint scar is visible in the paracentral cornea.

Three weeks later, the bandage contact lens was removed, the epithelial defect was entirely closed, and the corneal stroma was healed with minimal residual scarring (Figure 7 (Fig. 7)).

**Figure 8:**
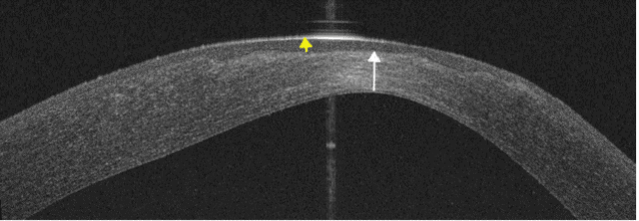
Anterior segment OCT of the left cornea 3 weeks post-operatively shows significant posterior elevation, apical stromal thinning (white arrow) (325 µm) and epithelial hypertrophy (yellow arrow) (92 µm) following surface healing.

The unaided visual acuity in the affected eye improved to 0.66 LogMAR, further improving to 0.4 LogMAR with pinhole. The anterior segment optical coherence tomography (OCT) showed a significant posterior ectasia, paracentral stromal thinning (white arrow) (233 µm) with associated epithelial hypertrophy (yellow arrow) (92 µm) to mask the stromal thinning and irregularity (Figure 8 (Fig. 8)), and Pentacam revealed maximum keratometry of 57.3 D.

Three months post-operatively, no recurrence of the plaque was noted, and the best spectacle-corrected visual acuity in the left eye was 0.3 LogMAR.

## Discussion

The present study comprises the first report of a calcific shield ulcer without AKC or VKC. This highlights the possibility of an alternative or additional element in the pathogenesis of shield ulcers in the absence of active inflammation, like potential friction between the palpebral conjunctiva and the ectatic corneal apex, which is often aggravated by eye rubbing, triggering the formation of epithelial micro-erosions and calcium deposition and resulting in the development of a calcific shield ulcer at the corneal apex, contrary to the conventional superior corneal shield ulcer. Given the thin epithelium over the cone in keratoconus patients [7], microabrasion is, theoretically, more expected in these eyes than in eyes with normal epithelial thickness. Nevertheless, mechanical friction cannot be the sole trigger for the development of shield ulcers since no similar ulcers have been reported in keratoconus patients so far. This stresses the importance of corneal surface protection in patients with corneal ectasias, irrespective of the absence of allergic eye disease.

Medical therapy includes a combination of topical anti-allergy and anti-inflammatory medication such as mast cell stabilisers, non-steroidal agents, corticosteroids and cyclosporin [3], topical antibiotics to prevent or treat secondary bacterial infection and lubricating drops, topical tacrolimus in refractory VKC [8], supratarsal injection of corticosteroids [9] or systemic immunosuppression [10]. In cases of non-healing shield ulcers despite adequate medical therapy, surgical interventions including temporary tarsorrhaphy [11], phototherapeutic keratectomy [12] and superficial keratectomy [4] are beneficial to achieve re-epithelialization. Moreover, a few studies have described successful use of amniotic membrane transplantation (AMT) to aid re-epithelialization in non-healing shield ulcers following debridement [13], [14].

Table 1 (Tab. 1) summarises two large case series on the management of shield ulcers. The majority of Grade 1 and 2 ulcers re-epithelialize with medical therapy alone [6], while out of all Grade 3 ulcers, only 1.7% achieved closure with medical therapy alone, as opposed to 100% re-epithelialization rate in eyes treated with surgical debridement and AMT [6].

Amniotic membrane facilitates corneal healing by reinforcing the adhesion between basal epithelial cells and enabling epithelial migration. In addition, it reduces scar formation by suppressing transforming growth factor B and the proliferation and differentiation of myofibroblasts in normal corneas [15].

Furthermore, the amniotic membrane reduces the inflammatory reaction by forming a protective barrier between the ocular surface and the tear film containing proinflammatory mediators [15]. However, fresh AMT is not readily available and requires a surgical theatre, which can delay treatment. Furthermore, it needs to be secured on the cornea with sutures, which carries suture related risk of infection [16]. 

In the presented case, the patient with a Grade 3 ulcer was treated with superficial keratectomy, in addition to medical treatment, in line with the literature evidence.

This is the first report of the use of a dry amniotic membrane, secured with a bandage contact lens, for the management of a non-healing shield ulcer. The amnion was absorbed within 2–3 weeks, following which the contact lens was removed. This new technology permitting, outpatient application of amniotic membrane on a cornea offers ample opportunities in the management of epitheliopathies refractive to medical treatment and nonhealing epithelial defects. Efficacy of dried amniotic membrane on healing persistent epithelial defect secondary to neurotrophic cornea, post-keratoplasty, severe dry eye, limbal stem cell deficiency due to Stevens-Johnson syndrome, glaucoma procedures, graft-versus-host disease and severe allergic reaction and infectious keratitis has been demonstrated [17].

Our patient achieved a significant improvement in visual acuity from LogMAR 1.06 to 0.3, which is better than the post-treatment visual acuity reported before [6]. This may be the consequence of the absence of an allergic inflammatory component in the presented case or of prompt surgical intervention with debridement and amniotic membrane use, which the authors advocate.

## Conclusion

Previously, shield ulcers have been exclusively reported in eyes with a history of AKC and VCK. We described a case of calcific shield ulcer in a patient with keratoconus without giant papillae or previous AKC/VKC, highlighting the potential for other contributing pathophysiological factors, like friction between the conjunctiva and a steep cornea. Based on our report, significant improvement in visual acuity is achievable with prompt surgical intervention and application of amniotic membrane in shield ulcers with persistent epithelial defects. Dry amniotic membrane provides an excellent alternative to conventional amniotic membrane transplantation in non-healing epitheliopathy with easy application and the possibility to use in the outpatient setting.

## Notes

### Video attachment

For a video of the surgical debridement of the shield ulcer’s calcified edge and insertion of dry amniotic membrane, see Attachment 1.

### Competing interests

The authors declare that they have no competing interests.

## Supplementary Material

Surgical debridement of the shield ulcer’s calcified edge and insertion of dry amniotic membrane

## Figures and Tables

**Table 1:**
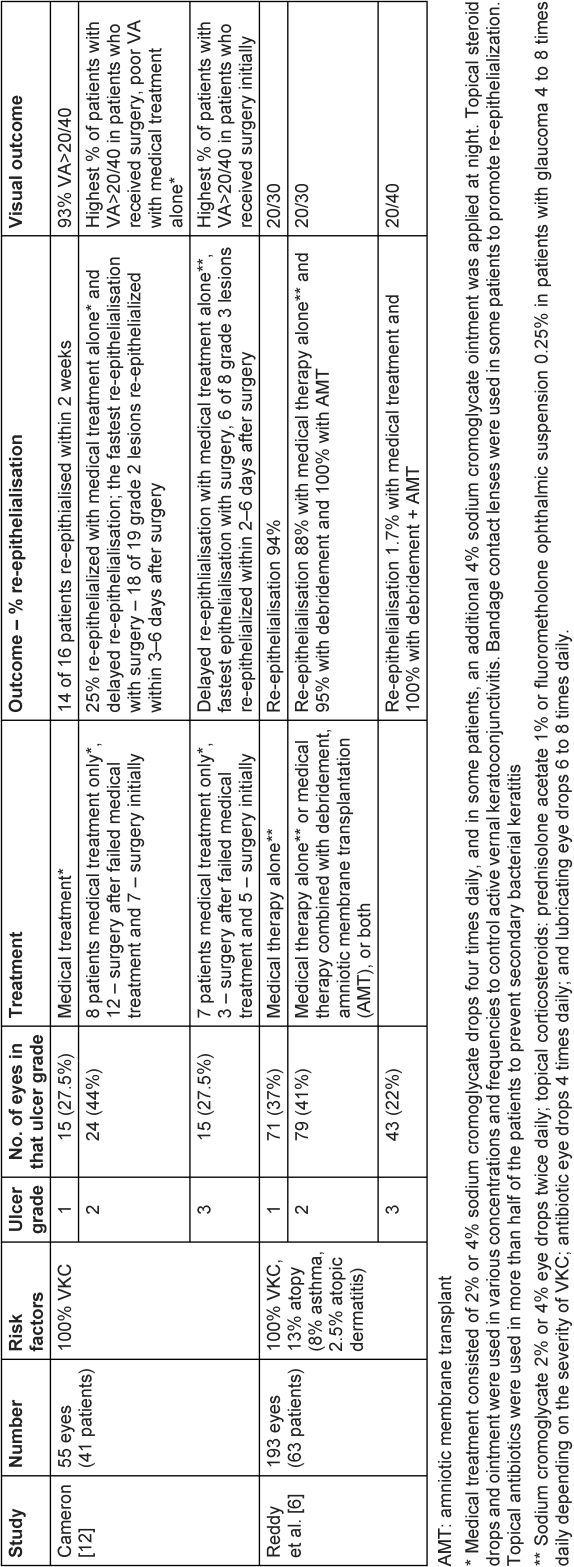
The two large retrospective case series on the management and outcome of shield ulcers
